# Hospital Work Abroad

**Published:** 1892-03-26

**Authors:** 


### HOSPITAL WORK ABROAD.

BY AN ENGLISH RESIDENT.

JOPPA (Jaffa) is supposed to be, next to Damascus, one of the oldest cities in the world.

It is peopled by the wild Bedouin Arabs, the more civilised Syrians, the dark Soudanese, German and Spanish Jews, European and Syrian merchants, and Turkish officials. During the winter and spring its population is greatly increased by numerous travellers and pilgrims from all parts of the world, who stay to rest for a few days in Jaffa on their way to Jerusalem. What a boon to them is the English hospital, where all who are sick and needy are received and cared for.

In the large, well-ventilated wards are gathered Hindoos, Moslems, Greeks, Roman Catholics, Nestorians, Jews, Copts, and Protestants, all thankful to find a haven of rest after travelling under the heat of the burning sun.

The hospital has very fine balconies and verandahs, where the patients delight to lie and watch the ships from afar sailing into the harbour, and to see the mail steamers getting farther and farther away, until there remains only a thin cloud of smoke in the far distance.

The Syrian children are very warm-hearted, and it is quite easy to win the love of the little sick ones by kindness, although at first they are very shy and timid.

How all the faces in the ward brighten when the "musiker" (a baby organ), is carried in, and a few simple Arabic hymns are sung, although only a few of the men can read, and still fewer of the women.

The Arabs are fond of boasting that Arabic is the richest language in the world (certainly it is one of the most difficult and complicated), and they are always grateful to English people for taking the trouble to learn to read their language.

The orange groves, which are the source of much of the beauty as well as the wealth of Jaffa, require to be constantly watered during the summer heat. The mist rising from the ground is a fruitful source of malaria; typhoid is also prevalent from tainted water, or the insanitary conditions of some of the houses in the crowded parts of the town.

The Syrians are very fond of riding, but owing to the sagacity of the Arab horses and the skill of the riders, they are rarely thrown; and as there are no factories there are seldom accidents, except from camel bites and sword cuts (? accidental).

Every Arab tribe has its Sheik (equal to chief), who is the lawgiver and doctor for his tribe. The ignorance and superstition of many of the Sheiks is lamentable; burning is one of their favourite remedies, so that many of the people are dreadfully disfigured by the scars of large burns.

Although there are many lepers in Jerusalem and other parts of Syria, there does not appear to be any leprosy in Jaffa. The Syrians are very brave in bearing pain, and many of them make splendid patients, with the exception that they are rather restless, the result of their wild, free life.

Ophthalmia in its worst forms rages in Jaffa, as in other Syrian towns; unfortunately, a favourite native treatment is to tie a thick linen handkerchief over the affected eye, and to leave it for days untouched and unwashed. Is it to be wondered at that blindness is so prevalent?

Sufferers from the effects of intemperance are unknown amongst the Moslems, and there is much less delirium when there is a high temperature than is usual with English patients.

I have been told that there is no asylum in the whole of Syria, as there would not be enough patients to fill one.

